# A novel hybrid model for species distribution prediction using probabilistic random forest, principal component analysis and genetic algorithm

**DOI:** 10.1371/journal.pone.0326122

**Published:** 2025-09-10

**Authors:** Taiwo A. Adekunle, Ibrahim K. Ogundoyin, Caleb O. Akanbi

**Affiliations:** Department of Computer Science, Osun State University, Osogbo, Nigeria; Sreenidhi Institute of Science and Technology, INDIA

## Abstract

Probabilistic Random Forest is an extension of the traditional Random Forest machine learning algorithm that is one of the frequently used machine learning algorithms employed for species distribution modeling. However, with the use of complex dataset for predicting the presence or absence of the species, It is essential that feature extraction is important to generate optimal prediction that can affect the model accuracy and AUC score of the model simulation. In this paper, we integrated the Genetic Algorithm Optimization technique, which is popular for its excellent feature extraction technique, to enhance the predictive performance of the PRF Model. a novel hybrid algorithm the genetically optimized probabilistic random forest algorithm, designed for predicting the distribution of *mastomys natalensis* in Nigeria. The model was also compared with existing models for dimensionality reduction with other optimization techniques, such as Principal Component Analysis, Grey Wolf, Optimizer optimized backpropagation neural network algorithm (GNNA), Butterfly Optimization Algorithm. These models were evaluated using four performance metrics, accuracy, the areas under curve, sensitivity, specificity, F1_score and precision. We also examined the spatial predictive distribution of the models. The results generated that the predictive performance of PRFGA, significantly improved compared to PRFPCA, GNNA and PRFBOA in predicting the presence or absence of *mastomys natalensis* with a presence only and pseudo-absence sample set. the PRFGA demonstrated a high predictive power in predicting the spatial distribution of the presence or absence of *mastomys natalensis* in Nigeria. The integration of the Genetic Algorithm optimization technique, stems from its renowned ability to address the specific challenges of data uncertainty and high-dimensionality reduction in feature extraction sets of SDMs, to enhance the performance of the PRF model.

## Introduction

Lassa fever, a viral hemorrhagic disease, poses significant public health threats in West Africa, particularly in Nigeria, where outbreaks have resulted in considerable morbidity and mortality. Despite efforts to control the disease, Lassa fever outbreaks continue to occur, highlighting the need for effective predictive models to enable early warning systems and targeted interventions. Existing predictive models in this regard have limitations in terms of accuracy, spatial resolution, and are not adapale to other zoonotic diseases. Therefore, there is a pressing need to develop an improved model in terms of accuracy and reliability and also create an adaptable framework on which other zoonotic diseases outbreak could be modelled, enabling proactive public health responses and reducing the impact of the diseases on affected communities, hence this research.

Species Distribution Modelling is an area of that encompasses several areas like machine learning and geo-computation, SDM has a major data repository to be a presence-only data modelling [1]. SDMs uses the geographical location (presence only) of the occurrence of a species with its corresponding climatic and environmental drivers, shown as the predictors to give a prediction of the species.

In recent times, several machine learning techniques have been used in the development of species distribution models ranging from machine learning models using ensemble(Random Forest, Probabilistic Random Forest, Maximum Entropy, and deep learning models, statistical models(Generalized Linear Models) [2–5] and capturing the relationship between the effect of climate change on species distributions, these also has impact on the predictive power all these algorithms used for the prediction of species with respect to climate change, that helps public health expert proffer solutions to predicted locations.

The wide range of machine learning techniques have generated quite lot of results, but few papers have been able to work on the optimization of these techniques as a form of cross validation, in addition to the existing models, the report from [6,7] uses an optimization method for dimensionality reduction to select the best and optimal variables or features for further machine learning prediction to generate optimal result, of introducing a novel hybrid model that utilized the Grey Wolf Algorithm with back propagation Neural Network [7], same as some reports from the use of butterfly optimization algorithm [8], sparrow search algorithm [9], principial component analysis [10].

Based on all these optimization techniques for dimensionality reduction, this study therefore introduces a unique method of using Probabilistic Random Forest (PRF), a machine learning model for noisy data, which is one of the reason for the choice of this algorithm [11–13], the PRF is an extension of the conventional random forest, that uses the features and labels, as a form of probability distribution instead of the deterministic, therefore, in this study we evaluated the model performance with Principal Component Analysis, Grey Wolf Algorithm, Neural Network, Support Vector Machine, and a spatial distribution map for the optimized PRFGA model.

Other machine learning algorithm like, MaxEnt is mostly used for presence-only dataset and its ability to handle small samples of data, and model complex nonlinear relationships [14], most MaxEnt models achieves a high accuracy in its prediction, and has a high tendency for overfitting when the model is not constructed correctly. SVMs are used for classification that has its strength in high dimensional dataset and also works relatively with small samples with minimization of the margin between the classes [15], the edge PRF has the ability to handle complex nonlinearities because it uses decision tree based ensemble structure, and works well with noisy datasets, it adds the ability to improve variable selection, which is very valuable in ecological data from several sources with many of its correlated predictors [16–17].

## Materials and methods

Probabilistic Random Forest uses the conventional RF classification-based algorithm, it is designed to improve the predictive accuracy of the conventional RF, by using its level of uncertainties in the input data (the GBIF data, and environmental and climatic merged dataset) as the input and using their information contexts [13]. Because of the large dataset there is need for optimal selection of features which in this context are predictors tat are used for the prediction of the presence or absence based on the presence location generated from the GBIF repository, therefore the feature selection procedure is important for dimensionality reduction to enhance the model performance, genetic algorithm uses a population-based evolutionary algorithm [14]. SDMs most times relies on presence-only data that captures observed locations but absence information not available, in the same vein most machine learning models require both the presence and absence data for effective training and testing, researchers resulted to the generation of pseudo-absence using target-group background points [1].

### Construction of the hybrid algorithm

In this study, we proposed a novel hybrid algorithm for the prediction of the distribution of *mastomys natalensis* in Nigeria, using the genetic algorithm optimized probabilistic random forest model. Majorly, we used the PRF to generate a model based on its selected features, then GA is then used to generate another feature, then this feature are used to develop another PRF model to generate and compare their accuracies.

**Fig 1:**
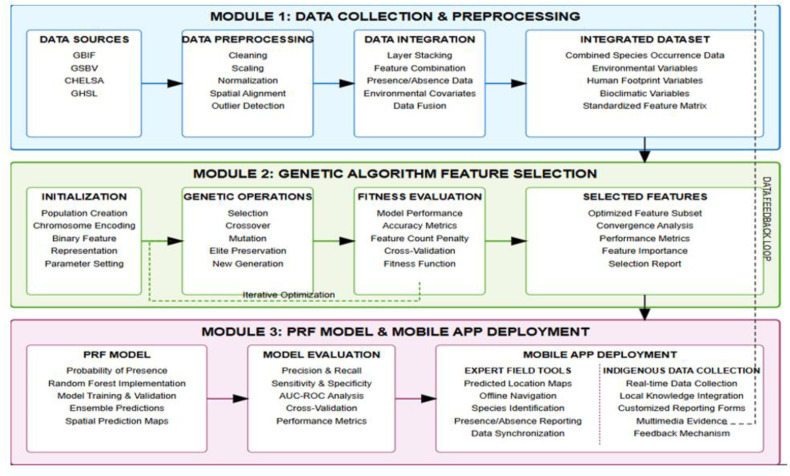
Model architecture.

Genetic algorithm uses heuristics search technique [14], which was influenced by the Darwins evolution theory, how animals and plays survive and transfer genes from one generations to another, which is also dependent on the number of populations, uses three major operation, selection, crossover, and mutation, the process of selecting the optimal features started by the initialization with our population size of 2000 samples after a pseudo-absence points have been generated, then a cross over probability is done, mutation and maximum generation was generated from the 2000 samples, of 20 population size, 10 number of generations, 0.7 crossover probability and 0.2 mutation probability, for each number of samples where there is a binary vector of 0 and 1, that is absence and presence, a set of subset of each features is created from the whole samples, then the evaluation of each population is done, for each features (p) in the samples(s), a feature (p) is selected, which is then returned as a set of another features, that goes into the PRF, for training the model on the selected features, and the fitness score in terms of its accuracy, AUC score, F1 Score is computed, which is selected based on their immediate contribution to the model performance. A detailed explanation of the model development is as shown in Fig 1, the model architecture, divided into three modules, the first model the data collection and preprocessing, the data sources(GBIF, GSBV, CHELSA, GHSL), preprocessed for removing NAs, normalization, scaling, and stacking, the stacked or integrated datasets ready for modelling, the second module is the genetic algorithm for feature selection, the third module is for the PRF Model from the selected GA features.

Algorithm 1. Pseudo code for PRFGA.

**Start:**

input GBIF, climatic and environmental data

output presence-absence of *mastomys natalensis*

**Process:**

**Step 1:** Initialization of Predictors:

Set sample size S, crossover probability CP, mutation probability MP and maximum number of Generation G_m_

**Step 2:** Generate Population:

Create an population {Y*i*,Y2, …,Y*P*} where each Yi is a binary vector of 2000 length (number of

predictors), representing a subset of features *Si* ∊ {p*i*, p2,.. p*n*}

**Step 3:** Evaluate Population:

For each individual Y*ji*,

select predictors P*ji*,

train a model PRF*i* on the selected features

compute fitness *F*(Y*i*) (e.g.,„ accuracy or Fl score).

**Step 4:** Repeat Step 1:3 until maximum generations is generated (10 generations)

**Step 5:** Return Best Predictors with PRF Model (PRFGA)

**Step 6:** Output = accuracy, AUC, F1 Score, of PRFGA test set

End

### Comparing PRFGA predictive performance with PRF and some commonly used species distribution techniques

Comparison was first made on the predictive performance of PRF and PRFGA with the postulate that PRFGA would outperform PRF and generate an optimal predictive performance. To achieve this aim, we downloaded presence only occurrence dataset for *mastomys natalensis* from the Global Biodiversity Information Facility (GBIF, http://www.gbif.org/) and duplicate values were removed at 5 km radius. These species are found in some parts of Nigeria to have been a major reservoir for the spread of Lassa fever in Nigeria influenced by the presence of some environmental and climatic variables, as well as population, a pseudo-absence data was generated based on the number of occurrence dataset, each occurrence data and pseudoabsence data is normalized with climatic dataset downloaded from (https://chelsa-climate.org/), soil dataset were downloaded from(https://zenodo.org/records/4558732), and population dataset from (https://human-settlement.emergency.copernicus.eu/), these dataset were merged and normalized to conform to the same geographical extent, abbreviations and full names of all the dataset as shown in Table 1.

**Table 1:** List of climatic and environmental variables and their sources.

Variable Type	Database Name	Variable Description	Layers	Source
climate	CHELSA	Bioclimatic	16	https://chelsa-climate.org/
Population	GHSL	Human Population	1	https://zenodo.org/records/4558732),
Soil	Soil Temp	Soil Temperature	48	https://zenodo.org/records/4558732),

As a first step, we constructed a model for the species using the PRF and PRFGA models respectively. For the *mastomys natalensis* host, we split the model into training and testing set of 80 for the training and 20% for the testing, the model predictive performance was then evaluated using the accuracy, sensitivity, specificity, AUC score, f1 score, precision.

Recent researches on SDM were used to make comparison of the model based on optimization techniques only namely, GNNA [7],PRFPCA [15], PRFBOA [17].

Following the methods adopted by bru et al [18], where 10 levels of environmental variables like the climate, soil and terrain were used a s predictor datasets, we set the species to fit four SDM optimization techniques, and a projection was made for predicting the presence of the species under the climate models and the model performance and prediction was evaluated, following the research by Zhang et al, [18,19] on the importance of dimensionality reduction of environmental variables for significant effect in the performance of the model, the response curve were generated, the genetic algorithm used a population size of 20, 10 number of generation, 0.7 crossover probability and 0.2 mutation probability, and the fitness function was defined to maximize the AUC, the best feature were selected for the development of the prf model,

### Comparison of spatial distribution predictions of *mastomys natalensis*

We also explored the comparison of the spatial distribution of the predictive performance using the metrics. The spatial distribution prediction gives the visual representation of locations that has been predicted by the model for the presence of *mastomys natalensis* in Nigeria, we applied PRF, PRFGA and two other optimization techniques used for SDM to predict the presence and absence distribution of mastoys natalensis under the current environmental and climatic conditions. We used native occurrence of *mastomys natalensis* to train the SDMs. The occurrence records of the *mastomys natalensis* in Nigeria were obtained from GBIF (http://www.gbif.org/), and the same for the climatic and environmental variables as depicted in Table 1.

## Results

### Comparison of predictive performance between PRF, and PRFGA

The five metrics consistently showed that PRFGA had a better predictive performance than PRF (Fig 1a–c), Specifically out of the fifty-five features(predictors) used in the model, PRFGA selected only twenty-two has been Significant and has high influence on the presence of *mastomys natalensis* in Nigeria, (Fig 1), with respect to the performance metrics, PRFGA has an accuracy of 0.8274 and PRF has an accuracy of 0.8010 and an AUC_Score of 0.9047 for the PRFGA compared to PRF model of 0.8783,

### Comparison of predictive performance between PRFGA and other optimization algorithms

Overall, the predictive performance of PRFGA was better than the Grey Wolf Optimization Algorithm with Back Propagation Neural Network (GNNA), Probabilistic Random Forest Principal Component Analysis (PRFPCA) and probabilistic Random Forest with Butterfly Optimization Algorithm (PRFBOA) for the prediction of *mastomys natalensis* (Figs 2–18) with a sample size of 2000, as well as the feature importance ranking generated from the PRF and PRFGA Models respectively.

**Fig 2:**
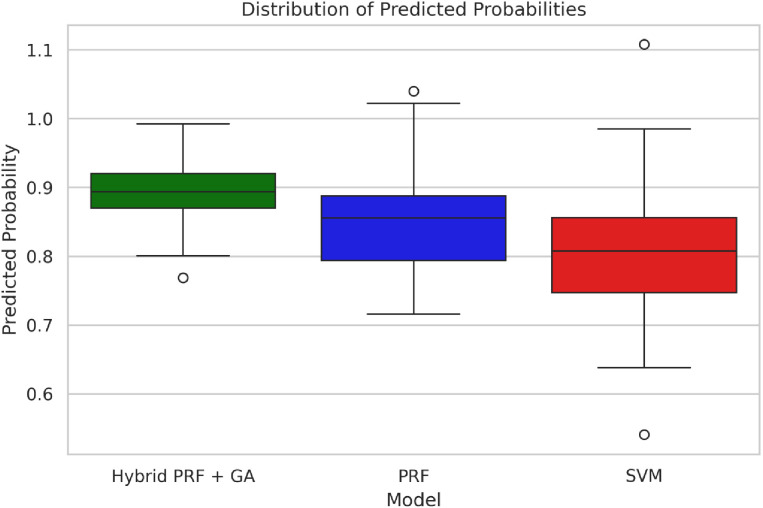
Comparison of predictive performance between PRF and PRFGA boxplot.

**Fig 3:**
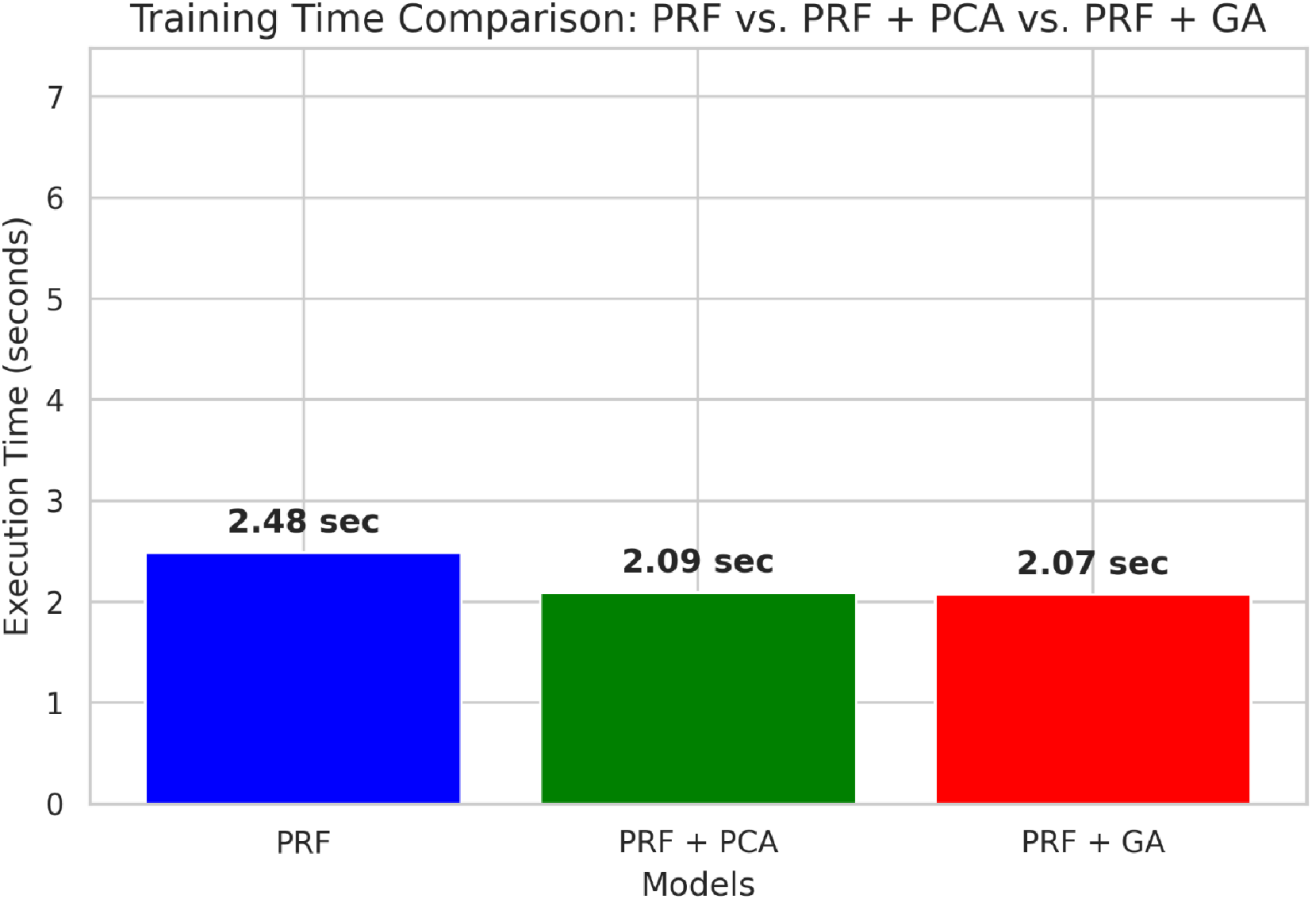
Training time comparison of the models respectively.

**Fig 4:**
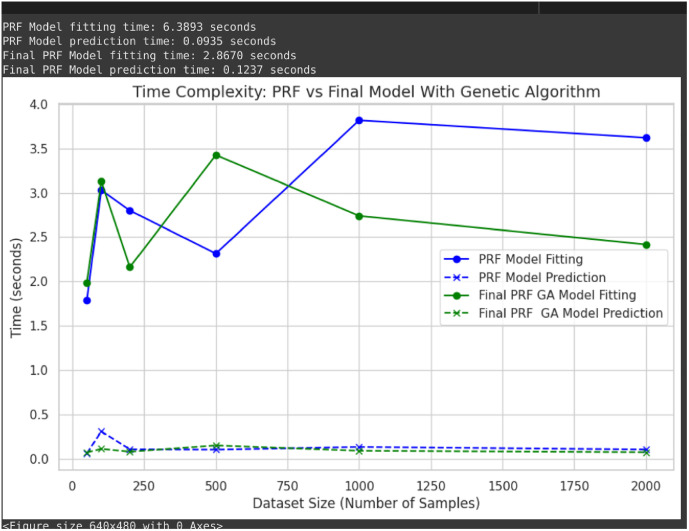
Time complexity of the model respectively.

**Fig 5:**
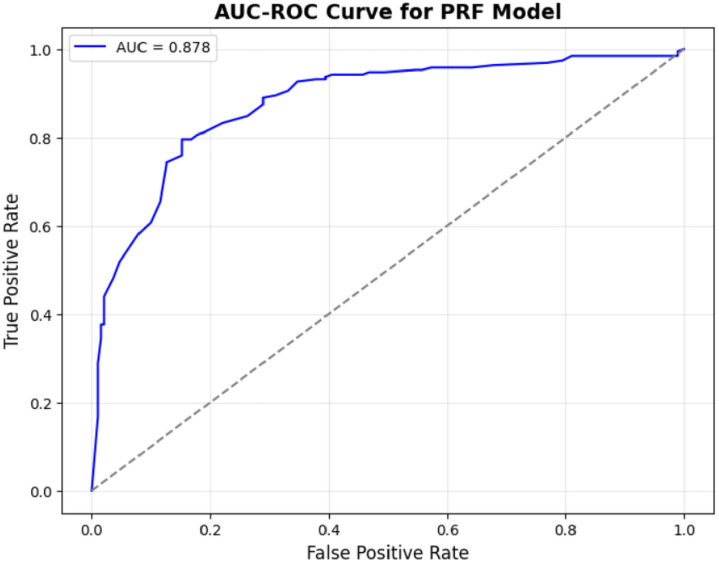
AUC-ROC curve for PRF model.

**Fig 6:**
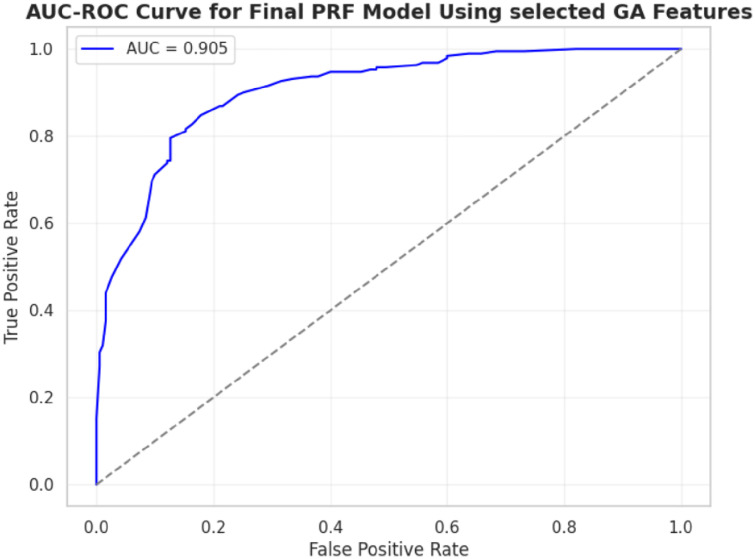
AUC-ROC curve for PRFGA model.

**Fig 7:**
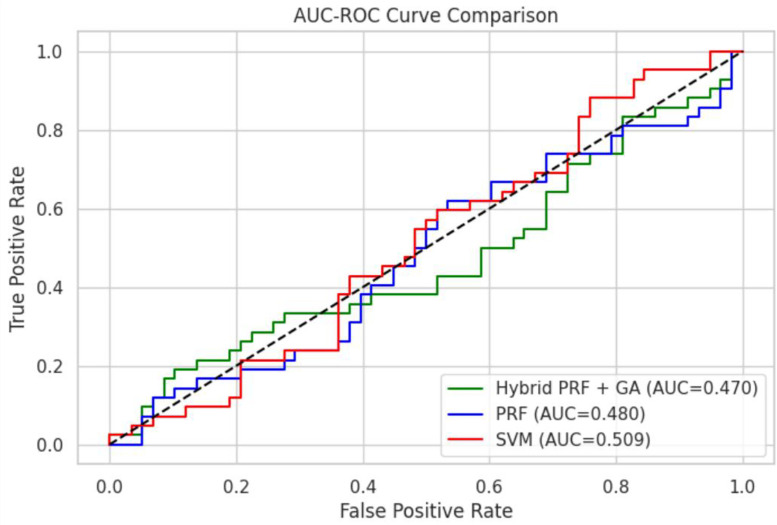
AUC comparison of Model between PRF and PRFGA.

**Fig 8:**
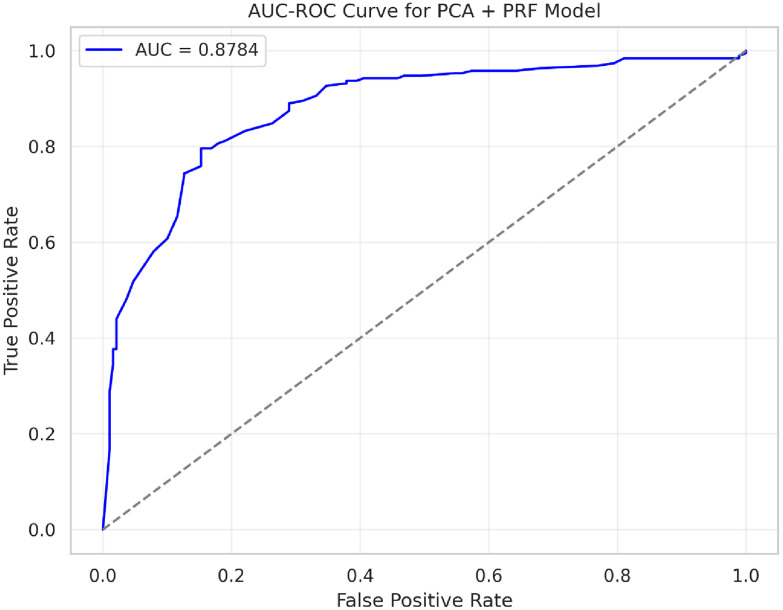
AUC-ROC curve for PRFPCA model.

**Fig 9:**
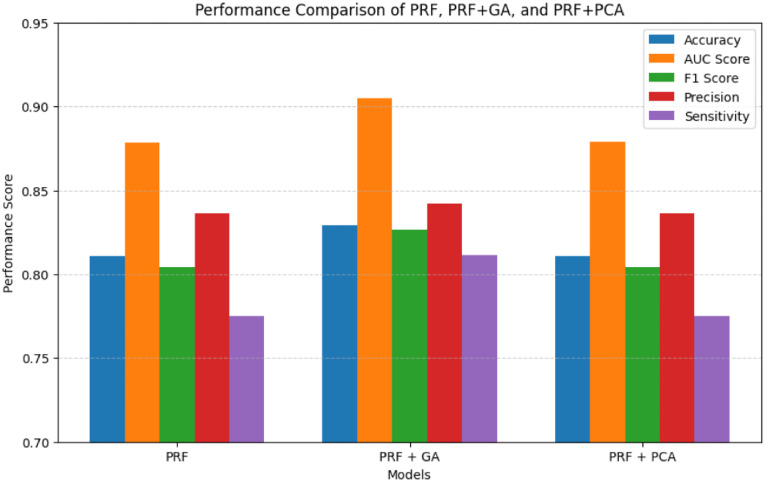
Model comparison of the two optimized models against the PRF.

**Fig 10:**
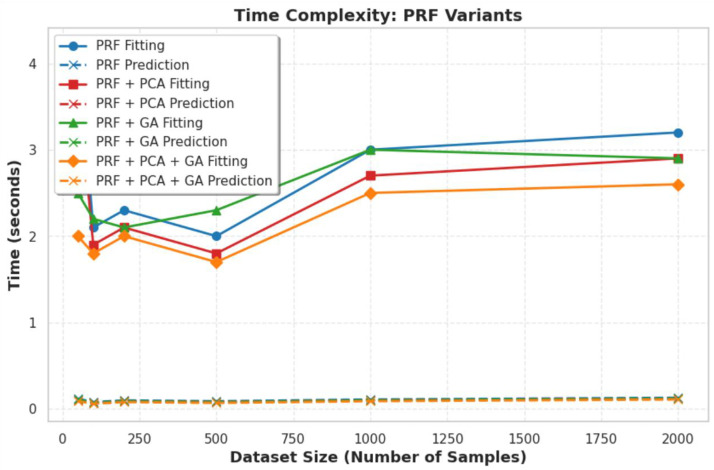
Running time(in seconds) of the model against samples.

**Fig 11:**
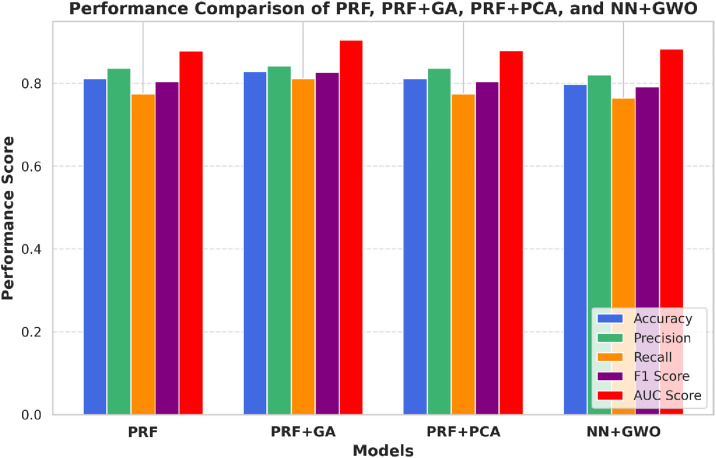
Model performance comparison of PRF, PRFGA,PRFPCA and GNNA.

**Fig 12:**
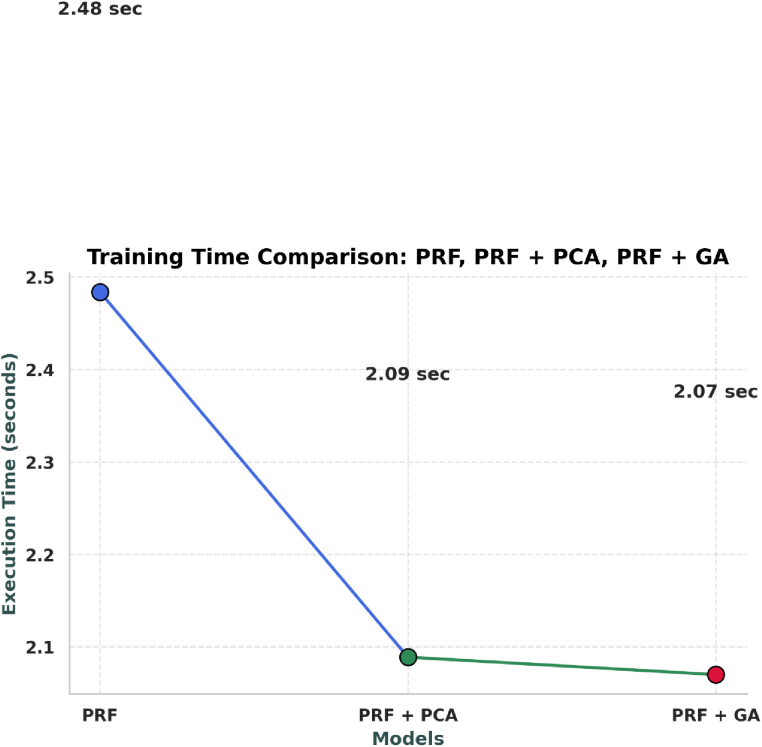
Training time comparison of PRF, PRFPCA, and PRFGA.

**Fig 13:**
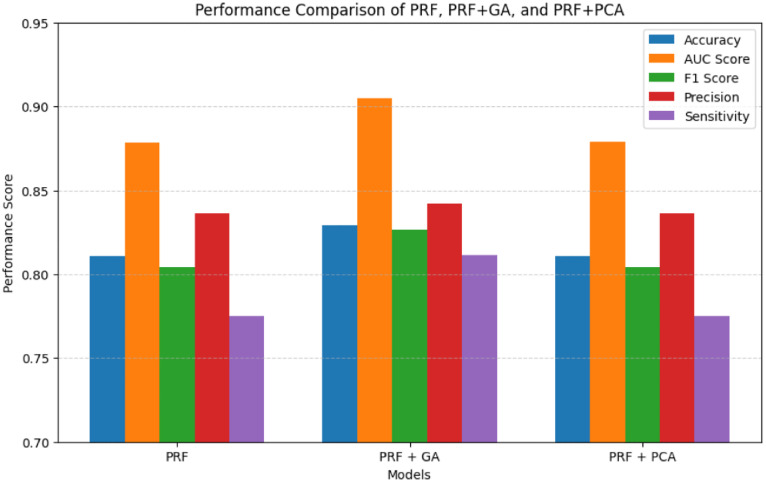
Performance metrics comparison of PRF, PRFPCA, and PRFGA.

**Fig 14:**
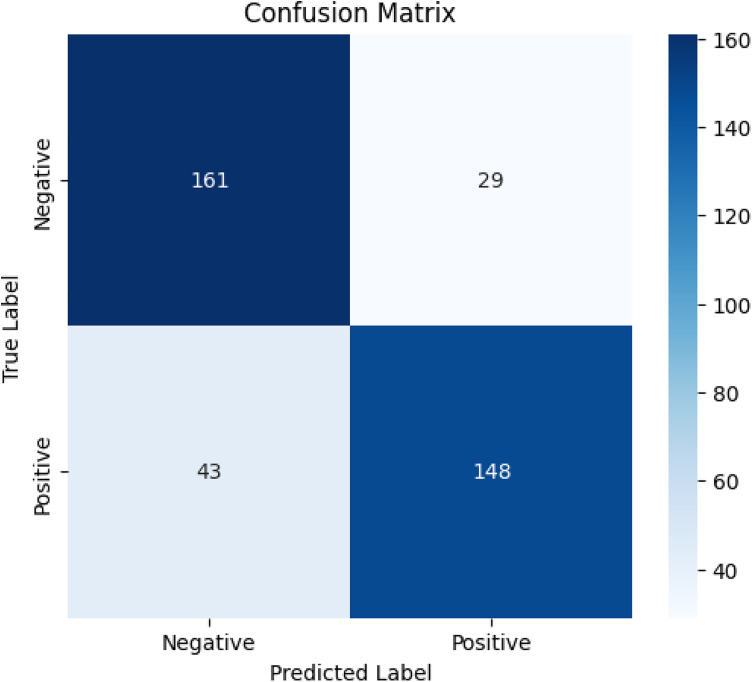
Confusion matrix for the PRF model.

**Fig 15:**
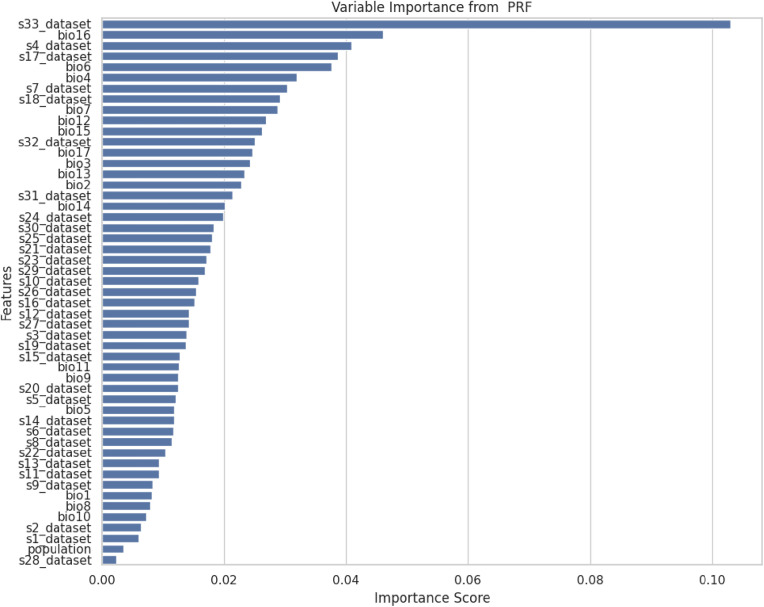
Variable importance for the PRF model.

**Fig 16:**
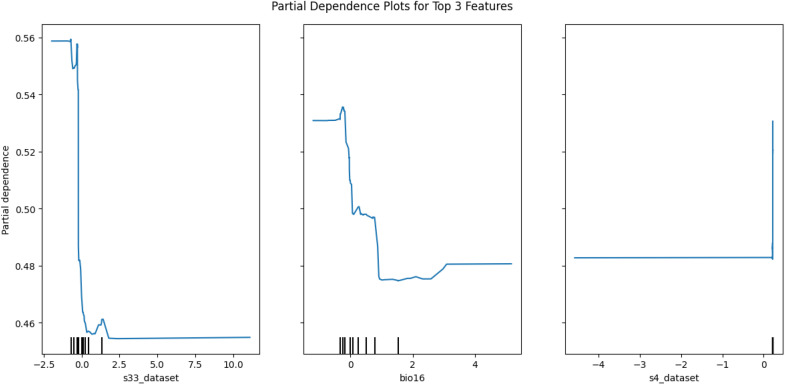
Partial dependence plot for the top 3 selected features.

**Fig 17:**
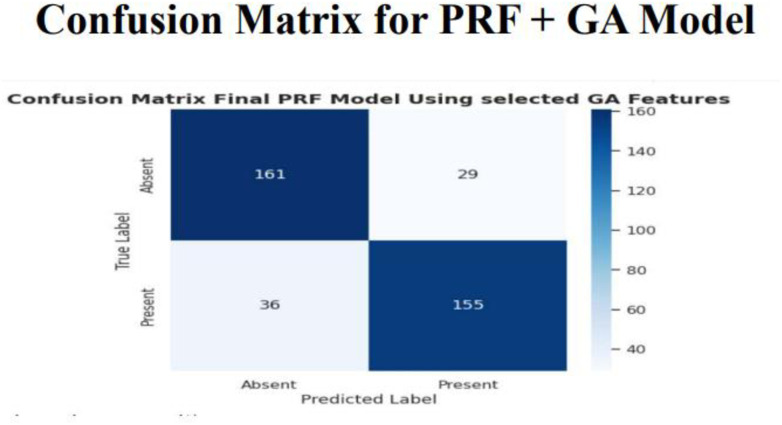
Confusion matrix for the PRFGA model.

**Fig 18:**
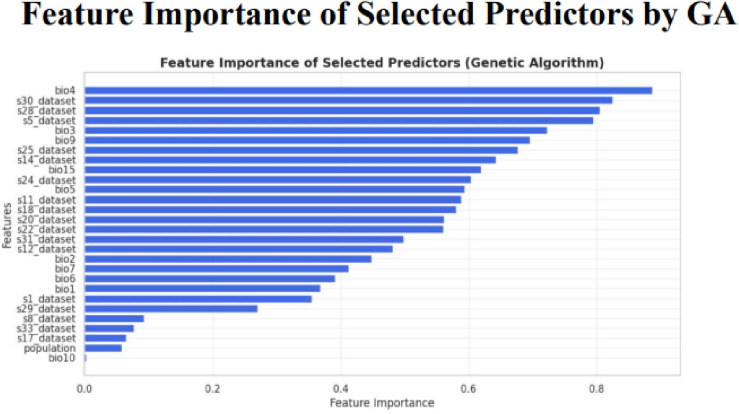
Feature importance of selected predictors by genetic algorithm.

### Cross validation of the hybrid algorithm

**Fig 19:**
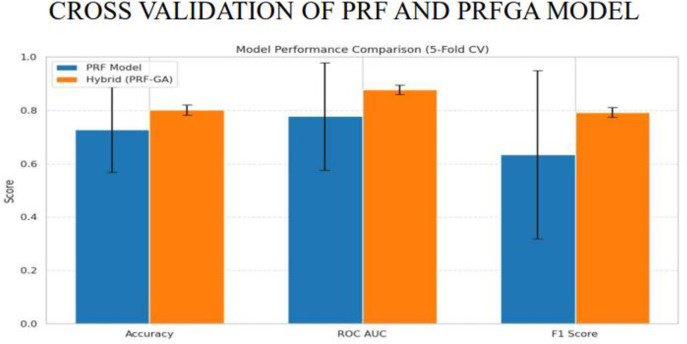
Cross validation of PRF and PRFGA models.

**Fig 20:**
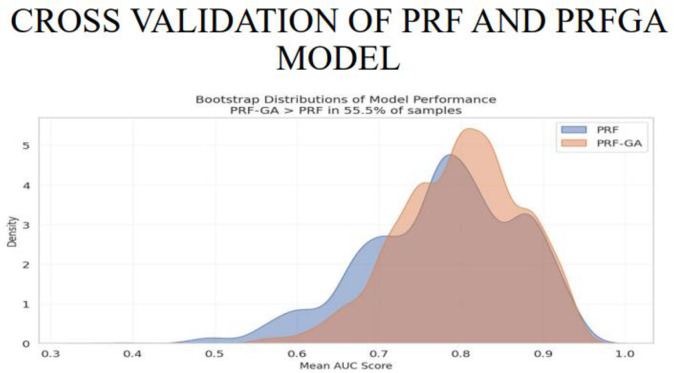
Bootstrap distribution of model performance of PRF and PRFGA model.

The cross validation of the hybrid model was implemented using the stratified K-fold cross validation with 5 folds, stratified k fold was used because each fold maintains the same proportion between the target classes, as there is a need to be consistent in the representation of the classes in both training and valuidation sets, 5 folds was used as this provides a good balance, Figs 19 and 20 gives the pictorial view of the validation.

**Fig 21:**
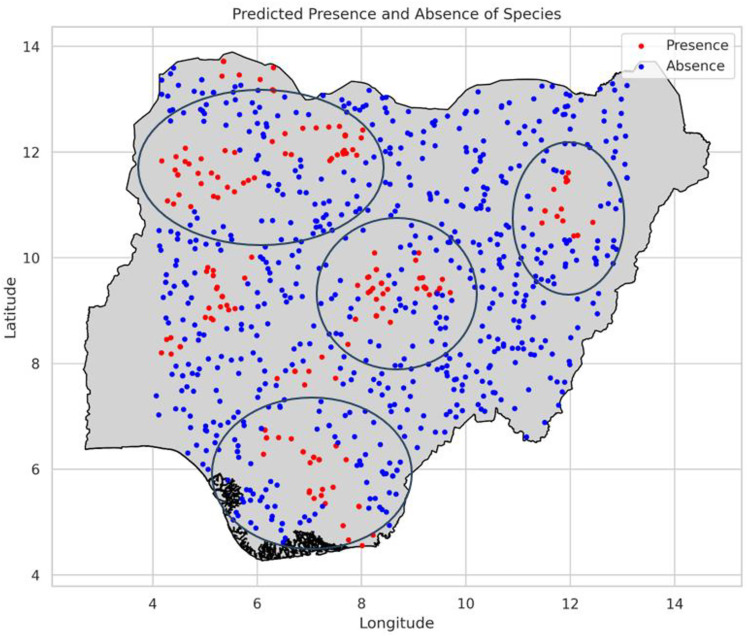
Model predicted sites for PRFGA.

**Fig 22:**
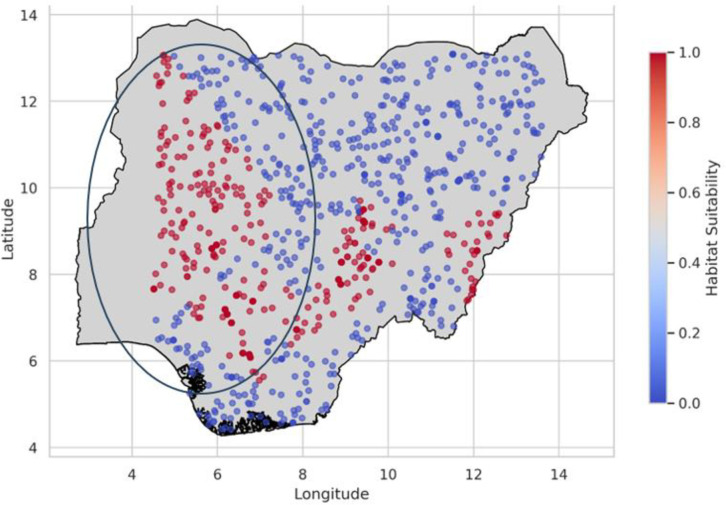
Model predicted sites for GNNA.

**Fig 23:**
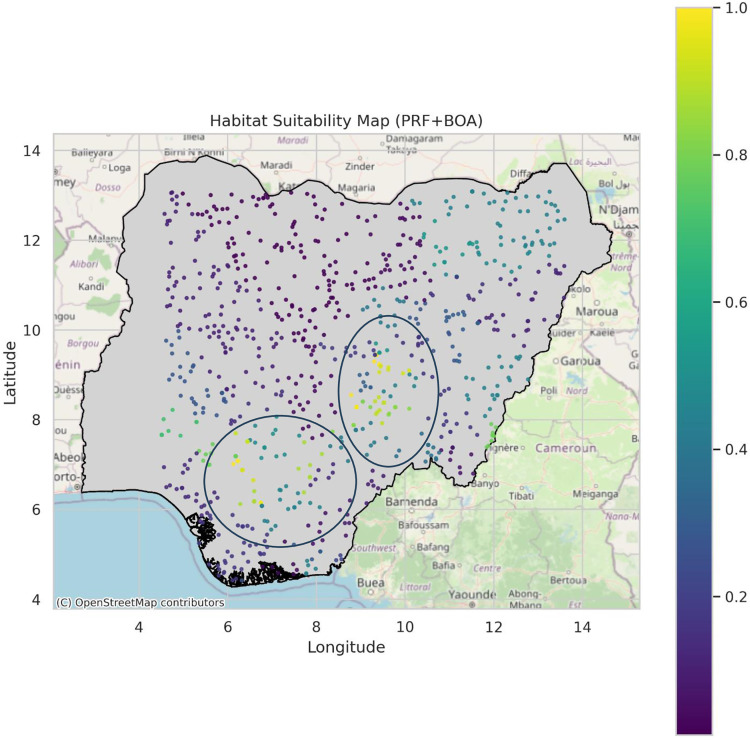
Model predicted sites for PRFBOA.

**Fig 24:**
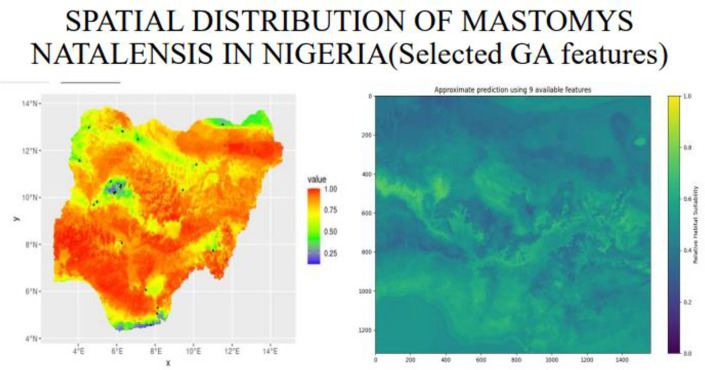
Spatial distribution of mastomys natalensis (selected GA features).

**Fig 25:**
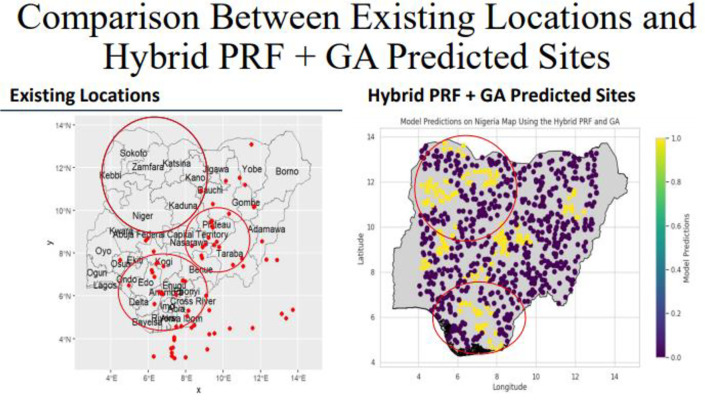
Comparison between existing locations and hybrid PRF + GA predicted sites.

The model predicted sites for the PRFGA, GNNA and PRFBOA are as shown in Figs 21–23, spatial distribution of Mastomys natalensis using the genetically optimized features is as shown in Fig 24, and a comparison of the model predicted sites of the hybrid model with the existing locations of lassa fever cases as shown in Fig 25.

## Discussion

The hybrid algorithm proposed, PRFGA demonstrated a higher improvement in the predictive performance over the traditional PRF as evidenced by the five distinct performance evaluation metrics. The development of an optimal predictive model plays a vital role in the prediction of species distribution models, and this research area iterates the importance of this research in SDM, it can only be observed that the accuracy score didn’t have a significant change used the hybrid method, compared to the AUC value that generated a substantial result, our comparative study of optimal model development reveals that the predictive capacity of PRFGA was better than the PRFPA and PRFBOA, which shoes the importance of Genetic algorithm in handling large datasets [8], merging Gas with other ensemble modelling techniques will give a more resilient and best suitable feature extraction approaches the PRF or handling noisy data, when combined together generated as a high potential to serve as a model construction for other ensembles models. The PRFGA was able to give a higher predictive performance because of its ability to explore the search space either necessarily or not, reduce accuracy performance problems and reduce the time complexity, and majorly the process of searching and finding important features for data mining helps in predicting the best possible solutions or predictors that affect the spread of lassa fever in Nigeria, with less predictive time and high model performance [20], the PRF takes into account uncertainties in the data, treating each predictors or features as a probability distribution function, instead the values, the importance of the level of uncertainty in the PRF rather than conventional RF both makes use of decision trees. The account of the level of uncertainty with the GA for feature extraction for complex dataset for optimization makes it a unique approach to solving SDMs [13].

The Distribution of *mastomys natalensis* like other species is greatly influenced by the climatic and non-climatic factors, among the climatic variables identified as the most influential is the temperature seasonality(BIO4), and this gives a reflection of how much temperature fluctuates over the year, in Nigeria, climatic variations is mainly driven by rainfall patterns and temperature seasonality is more pronounced in the north region, with hot days and cooler nights, the spatial distribution map generated suggested that the rodent rats are predominantly located in the southwestern and northeastern part of the country, this daily thermal oscillation could be a contributing factor for the suitable ecological niche for *mastomys natalensis*, the couther part of Nigeria maintain a stable temperature all year round with a combination of high rainfall and humidity, and this could be the effect of the temperature variability in the north and the moisture availability in the south, and the predicted location map by the hybrid model, reveals that *mastomys natalensis* have a higher likelihood in the southwest and northeast part of the country, the map illustrated a high probability in Osun, Ondo, Edo and Ogun states in the southwestern part and also Borno and Adamawa states in the northeastern part of the country, and the later representing the spatial distribution map, represents the model predicted habitat suitability reinforces this observation by highlighting similar regions indicating high probability, the striking overlap between the predicted locations and the spatial distribution confirms the models strength in identifying ecologically favorable zones for the species.

Among other influential environmental variables were the soil temperature of the warmest month (represented in this study as S30_DATASET). The soil temperature during the hottest month plays an essential role in microhabitat conditions, particularly in regulating burrow temperatures, influencing breeding cycles, and determining the availability of food resources for the rodents. In tandem, precipitation affects vegetation growth and ground cover, which in turn influences rodent shelter, movement, and food availability. The interactions among these variables create a complex, but identifiable ecological pattern that supports the proliferation of *M. natalensis*.

studies have demonstrated that climatic variables such as rainfall and temperature variability are strongly linked to rodent outbreaks and population dynamics, particularly in West African regions prone to Lassa fever [21,22].

In comparison with the Nigeria Center for Disease Control, the spatial patterns exhibit a strong alignment with historical and contemporary surveillance data, the red-to -orange gradients shows a strong alignment indicating areas of high probability significantly overlap with locations that are persistent with Lassa fever outbreaks as reported by NCDC, identified in Ondo, Edo, Buch, Taraba and Ebonyi states respectively, all within the probabilities of 0.75–1.0 on the ecological map.

In its 2025 epidemiological reports, the NCDC consistently identified Ondo and Edo States in the southwest, and Bauchi in the northeast, as the epicenters of Lassa fever incidence. These same regions fall within the red-circled areas of the spatial distribution map, underscoring a remarkable ecological-epidemiological concordance. Ondo State alone accounted for over 30% of the national case burden in early 2025, while Edo and Bauchi contributed 20% and 18% respectively—figures that match the model’s highest habitat suitability zones. This alignment reinforces the role of environmental and climatic factors in shaping rodent ecology and supports the use of ecological modeling as an early warning tool for Lassa fever surveillance.

## Conclusions

This research introduces an optimization technique into SDMs, and a hybrid algorithm that uses PRF and GA to improve the performance of the predictive model, compared with other optimization algorithm used with PRF, as proposed in this research paper has significantly improved. It can deduced that PRFGA has an excellent performance for prediction compare to other optimization techniques like Grey wolf Optimization with Neural Network and principal component analysis. Specifically, compared with PRF, the predictive performance of the hybrid algorithm PRFGA proposed in this paper is significantly improved, with the use of real-time environmental data, the proposed hybrid PRFGA model can be a good base learner for species distribution models to predict species response under future climatic scenarios and also a framework for other zoonotic diseases like Dengue and Ebola.

## Supporting information

S1 File Original model dataset. (CSV)
